# Referral for Ophthalmology Evaluation and Visual Sequelae in Children With Primary Brain Tumors

**DOI:** 10.1001/jamanetworkopen.2019.8273

**Published:** 2019-08-02

**Authors:** Yin Liu, Chenue Abongwa, Stephen Ashwal, Douglas D. Deming, Timothy W. Winter

**Affiliations:** 1 Department of Ophthalmology, Stanford University School of Medicine, Stanford, California; 2 Department of Pediatrics and Child Neurology, Loma Linda University Children’s Hospital, Loma Linda, California; 3 Department of Pediatrics and Hematology and Oncology, Loma Linda University Children’s Hospital, Loma Linda, California; 4 Department of Pediatrics and Hematology and Oncology, Children’s Hospital of Orange County, Orange, California; 5 Department of Pediatrics and Neonatology, Loma Linda University Children’s Hospital, Loma Linda, California; 6 Department of Ophthalmology and Neuro-ophthalmology and Pediatric Ophthalmology, Loma Linda University Medical Center, Loma Linda, California

## Abstract

**Importance:**

Visual impairment in children with brain tumors has received limited attention, as most pediatric neuro-oncology clinical trials neither require ophthalmologic evaluation on enrollment nor monitor effects of treatment on visual function during and after treatment.

**Objective:**

To investigate ophthalmology referral patterns for children with primary brain tumors, the prevalence of visual sequelae, and the association between tumor characteristics and vision-related diagnoses.

**Design, Setting, and Participants:**

This retrospective cohort study included 141 children with primary brain tumors treated at Loma Linda University Children’s Hospital and Eye Institute, a university-based tertiary referral center, between January 2013 and September 2017. Data analysis was completed in March 2019.

**Intervention:**

Comprehensive ophthalmologic evaluation for children with primary brain tumors.

**Main Outcomes and Measures:**

Percentage of patients with ophthalmology evaluation, prevalence of abnormal ophthalmic findings, and their association with tumor characteristics.

**Results:**

A total of 141 children (73 [52%] male; median [range] age, 7 [0-18] years) with primary brain tumors were enrolled in this study. Seventy-three patients (41 [52%] male; median [range] age, 8 [0-17] years) never had formal ophthalmologic evaluation. Sixty-eight patients (32 [48%] male; median [range] age, 7 [0-18] years) were evaluated by 1 of 4 board-certified, fellowship-trained pediatric and/or neuro-ophthalmologists for any visual impairment over a total of 222 visits. Five-year overall survival for patients who had eye examination was not significantly different from those who did not (mean [SD] survival, 78.3% [6.2%] vs 84.9% [4.7%]). Median (range) time from tumor diagnosis to initial ophthalmologic evaluation was 9 (0-94) months. Only 10 of 68 children (15%) presented with visual symptoms at tumor diagnosis, while 61 of 68 (90%) had abnormal findings on examination, including strabismus (41 [60%]), visual acuity impairment (37 [54%]), amblyopia (26 [38%]), papilledema (24 [35%]), visual field defects (13 [19%]), optic atrophy (12 [18%]), and keratopathy (10 [15%]). Strabismus occurred more frequently in patients with posterior fossa tumors (26 of 68 in posterior fossa vs 15 of 68 in other locations; *P* = .02). The presence of visual field defects in patients with no visual symptoms was 15% (9 of 58). Radiation was significantly associated with amblyopia (odds ratio, 4.5; 95% CI, 1.2-15.7; *P* = .02).

**Conclusions and Relevance:**

In this study, more than 50% of children with primary brain tumors were not referred for ophthalmologic evaluation. Although visual symptoms were uncommon, visual impairments occurred more frequently than previously reported. Ophthalmologic evaluation is recommended to identify and manage visual impairment and prevent permanent vision loss in children with brain tumors.

## Key Points

**Question:**

In children with brain tumors, what is the referral rate to ophthalmology and what is the prevalence of visual sequelae and their association with tumor characteristics?

**Findings:**

In this cohort study of 141 children with primary brain tumors, only 68 (48%) were referred for comprehensive eye examination. While only 15% of those referred had visual symptoms, more than 90% had a variety of visual impairments including both afferent and efferent diseases, with visual field defects being the most common impairment among patients without symptoms.

**Meaning:**

Ophthalmologic evaluation is recommended for early diagnosis of visual sequelae in children with brain tumors to prevent permanent vision loss.

## Teaser

This cohort study investigates the rate of ophthalmology referral, the prevalence of visual sequelae, and the association between tumor characteristics and ophthalmologic diagnoses in pediatric patients with primary brain tumors.

## Introduction

Brain tumors are the most common solid tumor and the second leading cause of cancer death in individuals aged 19 years and younger in the United States and Canada.^1^ Childhood brain tumor incidence varies by country from 1.12 to 5.14 cases per 100 000 persons, with the highest incidence in the United States.^1^ With improvements in diagnosis and treatments, 5-year overall survival is greater than 60%.^1,2^

Children with optic pathway and suprasellar tumors often have visual symptoms as a presenting feature,^3^ but tumors in other areas of the brain may also lead to permanent visual impairment,^4,5^ even without symptoms. One study^5^ at 2 large tertiary referral centers of 139 patients with posterior fossa tumors found symptoms in less than 50% of the cohort and reported that tumors with more aggressive growth patterns had significantly worse visual outcomes, including 17% with visual acuity less than or equal to 20/40. Another study^6^ of 182 patients with posterior fossa neoplasms found only 27% with ophthalmologic symptoms, and while esotropia was more common (29%), visual acuity was less than or equal to 20/40 in 10% of patients.

Brain tumors can alter the normal neuroanatomical structures of the visual system, leading to visual impairment and dysfunction.^4,5^ Visual impairment in childhood is associated with lifelong effects for children and their families^4^ and may affect self-perception, childhood development, education, driving eligibility, employment, and quality of life.^7,8^ As the survival of patients with brain tumors improves, untreated visual sequelae could be an important factor affecting quality of life.

Previous studies have focused largely on the effects of the tumor and/or treatment on vision and alignment^9,10^ with little attention to referral rate for ophthalmologic evaluation and the prevalence and association of visual impairment with tumor characteristics. This study adds to previous reports and recapitulates that prevention of permanent vision loss requires referral for ophthalmologic evaluation of children enrolled in clinical trials for brain tumors.

## Methods

This study was approved by the institutional review board at Loma Linda University. A waiver of informed consent was granted by the board given the retrospective nature of the study and minimal risk to patient care. Medical records of all children (age 0-18 years) with primary brain tumors from January 2013 to September 2017 at Loma Linda University Medical Center and Children’s Hospital were reviewed. Codes from the *International Classification of Diseases, Ninth Revision* (*ICD-9*) (191.0-191.9, 225.0-225.9, 227.3, 227.4, 237.0-237.9, 239.6, and 239.7) and *ICD-10* (C 71.0-71.9, D33.0-33.2, and D43.0-43.6) were used to identify patients with benign and malignant primary brain tumors under all categories. Patients were recruited from the Loma Linda University database regardless of enrollment status in clinical trials. Children with secondary brain tumors and pseudotumor cerebri were excluded. This study followed the Strengthening the Reporting of Observational Studies in Epidemiology (STROBE) reporting guideline.

At our institution, all children referred for ophthalmologic evaluation were seen by 1 of 4 board-certified, fellowship-trained pediatric and/or neuro-ophthalmologists. The following clinical data were collected for each patient: age at tumor diagnosis, sex, pathologic diagnosis, chief concern(s) at initial presentation (including visual concerns), tumor location, surgical resection classification, treatment modalities, and survival events. At each evaluation, the following data were collected when available: date of examination, chief concern(s), visual acuity, confrontation visual fields, slitlamp examination findings, dilated fundus examination, sensorimotor examination, cycloplegic refraction, visit assessment, and treatment plan. Visual acuity and confrontation visual field examinations were performed by a certified ophthalmology technician or a certified orthoptist in addition to a board-certified pediatric and/or neuro-ophthalmologist. Humphrey visual field testing, optical coherence tomography (OCT) of the retinal nerve fiber layer (RNFL), and color fundus photographs were obtained from cooperative patients.

### Statistical Analysis

Bivariate Pearson correlation coefficient (2-tailed), Spearman correlation coefficient, χ^2^ test, and *t* test were used to determine differences in frequency of each visual sequela in different groups identified by patient demographic characteristics, tumor characteristics, and treatments. Logistic regression with confidence intervals was calculated to determine likelihood of outcomes. Statistical analysis was performed using SPSS statistical software version 25 (IBM). Five-year overall survival was calculated using the Kaplan-Meier method with log-rank test to calculate differences between groups. Median survival time was determined using the reverse Kaplan-Meier survival method. A 2-sided *P* < .05 was considered statistically significant. The data analysis was completed in March 2019.

## Results

A total of 141 patients (73 [52%] male; median [range] age, 7 [0-18] years) with primary brain tumors were included (Table). There were 100 individuals who were newly diagnosed and 41 who were seen for follow-up evaluation (brain tumor was diagnosed prior to study enrollment). The most common tumor type was glioma, whereas other tumor types (eg, ependymoma and atypical teratoid rhabdoid tumors) occurred less frequently. There were more patients with parenchymal tumors and fewer with optic nerve and chiasm tumors (Table).

**Table.** Patient Demographic Characteristics at Diagnosis, Tumor Characteristics, and Survival

Variable	No. (%)	
	Patients With Eye Examination (n = 68)	Patients Without Eye Examination (n = 73)
Age, median (range), y	7 (0-18)	8 (0-17)
Sex		
Male	32 (47)	41 (56)
Female	36 (53)	32 (44)
Pathology		
Glioma	24 (35)	45 (62)
Medulloblastoma	9 (13)	5 (7)
Ependymoma	9 (13)	6 (8)
Atypical teratoid rhabdoid tumor	4 (6)	0
Choroid plexus carcinoma	5 (7)	1 (1)
Neuroepithelial tumor and pineoblastoma	3 (4)	3 (4)
Craniopharyngioma and prolactinoma	3 (4)	0
Germ cell tumor	3 (4)	0
Other	8 (12)	13 (18)
Tumor location		
Frontal lobe	4 (6)	12 (16)
Optic nerve and chiasm	19 (28)	6 (8)
Temporal, parietal, and occipital lobes	7 (10)	27 (37)
Posterior fossa and brainstem	37 (54)	28 (38)
Other	1 (2)	0
5-y overall survival, mean (SD), %	78.3 (6.2)	84.9 (4.7)
Follow-up time, median (range), mo	28 (1-180)	40 (1-217)

There were 73 patients (41 [52%] male; median [range] age, 8 [0-17] years; 45 newly diagnosed and 28 follow-up) without formal ophthalmologic evaluation despite 7 patients (10%) having visual symptoms including visual field deficits (2 patients [29%]), double vision (2 patients [29%]), blurry vision (2 patients [29%]), and abnormal gaze (2 patients [29%]). Others presented with nonvisual symptoms of hydrocephalus (31 of 73 [43%]), headaches (22 of 73 [30%]), and seizures (15 of 73 [21%]). There were no differences in age or sex distributions between patients with or without eye examinations (Table). Most patients with tumor location in the temporal, parietal, and 2 occipital lobes were not referred for ophthalmologic evaluation (27 of 34 [79%]). The 5-year overall survival for patients who had eye examination was not significantly different from those who did not (mean [SD] overall survival, 78.3% [6.2%]; 95% CI, 67%-92% vs 84.9% [4.7%]; 95% CI, 76%-95%; *P* = .58).

A total of 68 patients (48% of total; 32 [48%] male; median [range] age, 7 [0-18] years; 55 newly diagnosed and 13 follow-up) underwent ophthalmologic evaluation across 222 clinic visits. Median (range) time from diagnosis to ophthalmologic evaluation was 9 (0-94) months. Only 10 patients (15%) reported visual symptoms. Referral concerns at the initial ophthalmology evaluation included abnormities in vision (24 patients [35%]), gaze (23 patients [34%]), comprehensive eye examination (15 patients [22%]), and visual field defects (3 patients [4.4%]). Other concerns in 5 patients (7.4%) included droopy eyelid(s), increased tearing, eye pain, increased eye pressure, and eye bulging. Patients aged 3 years or younger were less likely to present with visual symptoms (Spearman ρ = 0.50, *P* < .001). Ophthalmologic evaluation identified 61 patients (90%) with any visual impairment.

Strabismus was diagnosed in 41 patients (60%); it was associated with amblyopia refractory to monocular occlusion, glasses, and/or surgery in 11 patients (27%). Strabismus was also strongly associated with visual acuity impairment (31 of 41 patients; ρ = 0.064; *P* < .001), refractive error (21 of 41; ρ = 0.326; *P* = .007), and amblyopia (22 of 41; ρ = 0.329; *P* = .006). Strabismus was more common in patients with posterior fossa tumors (26 of 41; ρ = 0.282; *P* = .02).

Decreased visual acuity was present in 37 patients (54%) in 1 or both eyes, of which 26 of 68 (38%) progressed to amblyopia. The causes included deprivation (ptosis, keratopathy and/or cataract) in 15 patients (58%), strabismus in 11 patients (42%) and refractive error in 11 patients (42%). Exposure keratopathy due to palsy of the seventh cranial nerve was found in 10 patients (15%), of whom 3 (30%) required aggressive management (tarsorrhaphy or corneal transplant). Radiation was significantly associated with amblyopia (15 of 26 [58%]; OR, 4.5; 95% CI, 1.2-15.7; *P* = .02).

Papilledema was diagnosed in 24 patients (35%) and associated with age older than 3 years (16 of 24 patients; ρ = 0.293; *P* = .02) and tumor location in the posterior fossa (8 of 24 patients; ρ = 0.328; *P* = .008). Resolution of papilledema was noted in 8 patients (33%) within 1 to 5 months (mean [SD], 2 [1.4] months) after surgery on initiation of chemotherapy. Optic atrophy was present in 12 patients (18%); in 5 patients atrophy had progressed from known papilledema, while in the other 7 it was found during initial ophthalmologic evaluation. Time from initial tumor diagnosis to discovery ranged from 1 month to 5 years.

Visual field defects were found by confrontation in 13 patients (19%) and by Humphrey visual field testing in 5 (7%); only 3 (4%) had symptoms. A total of 7 patients (10%) were able to perform Humphrey visual field testing. Visual field defect was the most common visual impairment among patients without visual symptoms (9 of 58 patients [15%]; ρ = 0.33; *P* = .007), although no association with any single tumor type or location was found.

## Discussion

In the studied cohort, fewer than 50% of children with primary brain tumors were referred for ophthalmologic evaluation. To our knowledge, this is the first study to report referral rates for ophthalmologic evaluation at a university-based, tertiary care center and provide comprehensive data on visual impairment. Ophthalmologic symptoms were similarly uncommon among those with or without ophthalmologic evaluation whose median age, sex distribution, overall survival, and follow-up time with treating clinicians were comparable (Table). When matched with previous, similarly sized cohorts, ophthalmologic symptoms in our population were less frequent, which likely limited referral rate for ophthalmologic evaluation.

Another possible explanation for low referral rates in our cohort is the difference in tumor characteristics between those with and without ophthalmologic evaluation. Fewer patients were referred for ophthalmologic evaluation with less aggressive tumor pathology; however, most patients with tumor location in the temporal, parietal, and occipital lobes were not referred for ophthalmologic evaluation (79%). Although these patients were under the care of the same neuro-oncologists, neurologists, and neurosurgeons who referred the patients for ophthalmologic evaluation, there is no standard guideline for referral for ophthalmologic evaluation that accounts for both tumor grade and location.

The prevalence of specific diagnoses in our cohort is comparable to results found in similarly sized cohorts. A study^9^ of 92 children with brain tumors found 15.2% with undiagnosed visual field defects, which is similar to the current report (19%). Description of papilledema in our cohort (35%) was also similar to rates ranging from 13% to 34% reported in previous studies.^3,11,12^ We note the exception is prevalence of strabismus (60%), which was nearly triple that found in previous reports (6%-29%).^3,11,12^ With more than 40% of patients with tumor location in the posterior fossa without ophthalmologic evaluation, the actual prevalence of strabismus may be higher.

Comprehensive ophthalmologic evaluations were performed by 4 board-certified, fellowship-trained pediatric and/or neuro-ophthalmologists. All visual impairments were recorded, irrespective of consideration for relation to tumor characteristic, which likely explains why our study’s rates of visual impairment exceed those of previous reports. Armstrong et al^13^ reported a 15-year 18% cumulative index of blindness in 240 childhood low-grade glioma survivors. Another study of 200 children with brain tumors reported a 38% incidence of visual difficulties at any point in their course of treatment.^8^ Although our report of 90% visual impairment seems high in comparison, previous reports of visual impairment in pediatric brain tumors have omitted publication of referral rates for ophthalmologic evaluation. Review of the current large, multicenter, cooperative clinical trials for pediatric brain tumors confirms no standard to refer for ophthalmologic evaluation.^14^ Given a low percentage of patients with visual symptoms in our cohort without ophthalmologic evaluation, tumor management may have been prioritized over ophthalmologic evaluation despite evidence that treatment may affect vision.^15^ Additionally, the absence of a standard to refer for ophthalmologic evaluation may explain underestimated prevalence of visual impairment in previous reports.

Both afferent and efferent visual impairment in childhood may result in lifelong effects for the child and the family,^4^ and may also affect self-perception, childhood development, education, driving eligibility, employment, and quality of life,^7,8^ especially as the overall survival of patients with brain tumors continues to improve. Our report highlights that neither clinicians nor caregivers can rely on a patient’s report of visual symptoms, especially patients aged 3 years or younger. A referral for ophthalmologic evaluation is recommended for all patients enrolled in clinical trials to treat brain tumors, which includes age-appropriate visual acuity, visual field, and ocular motility evaluation; intraocular pressure; dilated fundus examination; and refraction. Further evaluation with OCT of the RNFL and ganglion cell complex, fundus photographs, and Humphrey visual fields may also be indicated.

Repeated evaluation may be helpful for tumor surveillance, especially for papilledema,^4^ which may be monitored with OCT of the RNFL and ganglion cell complex. The effect of papilledema on the visual system can also be measured by formal visual field testing. In one study, 7% of patients were diagnosed with recurrent disease based on abnormal visual field testing,^9^ while another study showed association between tumor relapse and visual field defects.^16^ In the latter, both patients with persistent papilledema eventually had tumor progression; 1 also had worsening homonymous hemianopia associated with tumor recurrence.

### Limitations

This study has limitations. We studied the visual sequelae of children with primary brain tumors who were referred for ophthalmologic evaluation without a standard protocol. There may also be a few patients with ophthalmologic evaluation outside our institution whose visual impairments have not been evaluated in this cohort, although we estimate this to be less than 5% given complexity of coexisting neurologic conditions in this population whose insurance constraints require in-network referral for pediatric ophthalmologic evaluation. Additionally, the population studied was less than half of the total cohort, which subjects the conclusions in this study regarding the prevalence of visual sequelae in children with primary brain tumors to confounding and referral among other biases. A large, prospective study and/or required ophthalmologic evaluation for all children enrolled in clinical trials to treat brain tumors may further clarify associations of visual sequelae and tumor characteristics.

## Conclusions

In this study, more than 50% of children with primary brain tumors were not referred for ophthalmic evaluation. The time for a multidisciplinary, comprehensive approach to the diagnosis and management of visual impairment in pediatric patients with brain tumors is now. Given significant advances in technology, OCT analysis of the RNFL and ganglion cell complex in children as young as 3 years routinely complement a thorough pediatric and/or neuro-ophthalmology evaluation at our institution. These and other tests alert the patient, their family, and other health care professionals to details regarding microscopic, neuroanatomical changes in the retina and optic nerve before loss of function in some cases. As such, cure may be defined as prevention of permanent vision loss.
